# Targeting Angiogenesis in Biliary Tract Cancers: An Open Option

**DOI:** 10.3390/ijms18020418

**Published:** 2017-02-15

**Authors:** Valeria Simone, Oronzo Brunetti, Luigi Lupo, Mario Testini, Eugenio Maiorano, Michele Simone, Vito Longo, Christian Rolfo, Marc Peeters, Aldo Scarpa, Amalia Azzariti, Antonio Russo, Domenico Ribatti, Nicola Silvestris

**Affiliations:** 1 Operative Unit of Internal Medicine, Hospital “F.Ferrari”, 73042 Casarano (Le), Italy; valeriasimo@gmail.com; 2 Medical Oncology Unit, Cancer Institute “Giovanni Paolo II”, 70124 Bari, Italy; dr.oronzo.brunetti@tiscali.it; 3 Department of Emergency and Organ Transplantation, Institute of General Surgery and Liver Transplantation, University of Bari, 70124 Bari, Italy; luigig.lupo@gmail.com; 4 Department of Biomedical Sciences and Human Oncology, Unit of Endocrine, Digestive and Emergency Surgery, 70124 Bari, Italy; mario.testini@uniba.it; 5 Department of Emergency and Organ Transplantation, Operating Unit of Pathological Anatomy, “Aldo Moro” University, 70124 Bari, Italy; eugenio.maiorano@uniba.it; 6 Surgical Oncology Unit, Cancer Institute “Giovanni Paolo II”, 70124 Bari, Italy; misimone.67@gmail.com; 7 Medical Oncology Unit, Hospital of Taranto, 74010 Taranto, Italy; vito.longo79@tiscali.it; 8 Phase I–Early Clinical Trials Unit, Oncology Department, Antwerp University Hospital & Center for Oncological Research, 2650 Edegem, Belgium; christian.rolfo@uza.be; 9 Oncology Department, Antwerp University Hospital, 2650 Edegem, Belgium; marc.peeters@uza.be; 10 ARC-NET (Applied Research on Cancer-Network) Research Centre, University of Verona, 37134 Verona, Italy; aldo.scarpa@univr.it; 11 Department of Diagnostics and Public Health, Section of Pathology, University of Verona, 37134 Verona, Italy; 12 Preclinical and Clinical Pharmacology Unit, Cancer Institute “Giovanni Paolo II”, 70124 Bari, Italy; a.azzariti@oncologico.bari.it; 13 Department of Surgical, Oncological and Oral Sciences, Section of Medical Oncology, University of Palermo, 90144 Palermo, Italy; antonio.russo@usa.net; 14 Department of Basic Medical Sciences, Neurosciences and Sensory Organs, University of Bari Medical School, 70124 Bari, Italy; domenico.ribatti@uniba.it; 15 Cancer Institute “Giovanni Paolo II”, 70124 Bari, Italy

**Keywords:** biliary tract cancers, angiogenesis, vascular endothelial growth factor, monoclonal antibodies, tyrosine kinase inhibitors

## Abstract

Biliary tract cancers (BTCs) are characterized by a bad prognosis and the armamentarium of drugs for their treatment is very poor. Although the inflammatory status of biliary tract represents the first step in the cancerogenesis, the microenvironment also plays a key role in the pathogenesis of BTCs, promoting tumor angiogenesis, invasion and metastasis. Several molecules, such as vascular endothelial growth factor (VEGF) and fibroblast growth factor (FGF), are involved in the angiogenesis process and their expression on tumor samples has been explored as prognostic marker in both cholangiocarcinoma and gallbladder cancer. Recent studies evaluated the genomic landscape of BTCs and evidenced that aberrations in several genes enrolled in the pro-angiogenic signaling, such as FGF receptor-2 (FGFR-2), are characteristic of BTCs. New drugs targeting the signaling pathways involved in angiogenesis have been tested in preclinical studies both in vitro and in vivo with promising results. Moreover, several clinical studies tested monoclonal antibodies against VEGF and tyrosine kinase inhibitors targeting the VEGF and the MEK/ERK pathways. Herein, we evaluate both the pathogenic mechanisms of BTCs focused on angiogenesis and the preclinical and clinical data available regarding the use of new anti-angiogenic drugs in these malignancies.

## 1. Introduction

Biliary tract cancers (BTCs) comprise cholangiocarcinoma (CCA), arising from the epithelial lining of the bile ducts, and gallbladder cancer (GBC). CCA can be subclassified into intrahepatic (iCCA) and extrahepatic cholangiocarcinoma (eCCA), depending on the origin from the biliary tree within the liver or outside the liver parenchima [1]. eCCA also comprises perhilar CCA (pCCA), which is a subset involving the bifurcation of the ducts [2]. iCCA, pCCA and eCCA are distinct entities that require specific management. They are classified by different WHO classification and AJCC/UICC TNM staging and are linked to distinct risk factors [3,4]. Furthermore, iCCA and eCCA are characterized by different pathological features, with iCCA predominantly mass-forming and pCCA often periductal-infiltrating. Most CCA and GBC are adenocarcinoma and are graded according to the percentage of glandular tissue [4].

BTCs represent 10%–15% of primary liver cancers and CCA is the second most common primary liver tumor worldwide after hepatocellular carcinoma (HCC) [5]. Prognosis of advanced and metastatic BTCs is poor with a five-year survival rate of about 2% for stage IV intrahepatic, extrahepatic bile duct and gallbladder cancer [6]. Management of early stage and locally-advanced BTCs includes surgery and chemo-radiotherapy while systemic chemotherapy is the main treatment of metastatic disease. The cisplatin/gemcitabine combination represents the standard of care and the ABC-02 trial demonstrated that the combination therapy offers a median survival of 11.7 months as compared with 8.1 months for the gemcitabine-only group [7]. A second-line treatment demonstrated a limited value in this setting of patients, with a response rate of 10.2% in a retrospective study enrolling 174 patients pre-treated with the cisplatin/gemcitabine combination [8].

Pathogenesis of BTCs involves several mechanisms whose investigation could be crucial in the identification of new potential therapeutic targets. Experimental data suggest that a chronic inflammatory status in the biliary tract promotes the malignant transformation of cholangiocytes [9,10]. In particular, cytokines and growth factors assume the main role in promoting the cancerogenesis in the biliary tract through the damage of mismatch repair genes, tumor suppressor genes and oncogenes. Indeed, cytokines activate the nitric oxide synthase favoring the production of reactive nitrogen oxide species that interact with DNA.

The microenvironment also plays a key role in the pathogenesis of BTCs promoting the tumor angiogenesis, invasion and metastasis. Cancer-associated fibroblasts promote tumor growth and angiogenesis through the production of several molecules, including vascular endothelial growth factor (VEGF), fibroblast growth factor (FGF), and interleukin-6 (IL-6) [11]. Moreover, the production of VEGF-A, IL-10 and transforming growth factor beta (TGFβ) causes the polarization of macrophage toward the pro-angiogenic phenotype M2 [12].

The aim of this review is to describe the role of angiogenesis in the pathogenesis of BTCs. These tumors, especially CCA, are often grey-white scyrrous masses with a poor vascularization, unlike hepatoarcinoma. However, recent studies focused on the role of pro-angiogenic pathways in BTCs pathogenesis. Then, the role of anti-angiogenic drugs is debated because of not entirely encouraging results from some clinical studies. After a preliminary assessment of the pathological and genomic data, we will analyze the preclinical and clinical data in the anti-angiogenic approach in the therapeutic management of these patients.

## 2. BTC-Associated Angiogenesis and Lymphangiogenesis: From Pathological Features to Genic Regulation

Angiogenesis and lymphangiogenesis are the biological processes that lead to the formation of new vessels from pre-existing vascular and lymphatic vessels. In malignancies, tumor cells produce or lead the microenvironment to the production of pro-angiogenic and lymphangiogenic signals able to recruit and expand endothelial cells [13]. Angiogenesis and lymphangiogenesis have been investigated in several gastrointestinal tumors [14,15,16,17]. Microvessel density (MVD) correlate with cancer progression, metastasis, and prognosis in GBC [18], iCCA [19], and hilar CCA [20]. VEGF was expressed in 53.8% and 59.2% of 236 iCCA and eCCA, respectively, even if it was significantly associated with intrahepatic metastasis only in iCCA [21,22]. Furthermore, VEGF-A expression was evaluated in 111 iCCA cases and compared between hilar and peripheral iCCA. The authors observed that VEGF-A was overexpressed in peripheral cholangiocarcinoma (69% vs. 25%, *p <* 0.0001) and correlated with increased vascular density [23]. This finding suggests a potential different imaging of hilar and peripheral iCCA and a possible best response of peripheral iCCA to anti-angiogenic therapies. VEGF expression in eCCA was associated with peritoneal recurrence and shorter survival [24]. Moreover, VEGF was significantly associated with angiogenesis but not with patients survival [25] and prognosis [26] in GBC.

VEGF-A was express in around 80% of GBC, with 56.3% of 84 patients with a high expression, resulting an independent prognostic factor of survival [27]. A meta-analysis of 102 different immunohistochemical biomarkers, comprising epidermal growth factor receptor (EGFR), c-erb-B2 and VEGF-A [28], demonstrated that VEGF-A resulted more expressed in iCCA respect to eCCA (RR: 2.78, 95% CI 1.69–4.58). Probably, the liver pro-angiogenic microenvironment may influence this difference [29]. Tumor-associated macrophages, polarized toward the phenotype M2 by several cytokines present in the tumor microenvironment, activate angiogenesis process through the production of VEGF [30,31]. Conversely, interferon-γ (IFN-γ) inhibits the differentiation of macrophages and favors the phenotype M1. When IFN-γ was intratumorally administered in a GBC xenograft model subcutaneously injected with a human GBC cell line. MVD and VEGF concentration were significantly reduced [32].

Another group assessed the role of VEGF-D both in GBC cell lines and in a xenograft mouse model. An inhibitory effect both on proliferation and invasiveness was observed in vitro by using a VEGF-D siRNA and confirmed in the subcutaneous and orthotopic xenograft tumors [33].

Moreover, lymphangiogenic VEGF-C and -D resulted overexpressed in a group of 50 GBC (>60%, in 32 and 31 of 50 patients, respectively) and their high expression correlated with lymph node metastasis via the nuclear factor (NF)-κB pathway [34,35], as observed in a small cohort of 20 patients.

In GBC, VEGF-C expression and MVD have been correlated with clinical outcomes and pathological aspects. Sixty-three percent of 52 GBCs overexpressed VEGF-C protein by immunohistochemistry. It was observed that the overexpression of VEGF-C was associated with both worse outcomes and a higher incidence of lymph node metastasis, thus suggesting the role of VEGF-C in promoting tumor progression via lymphangiogenesis [36].

VEGF-C expression was significantly correlated with lymphatic vessel involvement, lymph node metastasis, and worse outcomes after operation (all *p <* 0.001), but not with MVD. By the Cox regression model, lymphatic vessel involvement emerged as an independent prognostic parameter. These results suggest that VEGF-C may play a role in tumor progression and lymph node metastasis in human GBC.

Beyond the effect of VEGF, the interplay of VEGF with angiopoietin (Ang)-1/2 and thrombospondin (TSP-1) exerts a relevant pathogenic role in CCA. Tang et al. observed that VEGF and Ang-2 might play a pro-angiogenic role, while TSP-1 may play an inhibitory role [21]. Ang-2 cooperates with Ang-1 in the regulation of endothelial quiescence binding its receptor Tie-2. Its overexpression has been related to the neovascularization process in several tumors. Voigtlander et al. observed that high circulating levels of Ang-2 in CCA patients. The study demonstrated that this serum marker allows the distinction of patients with CCA from those with biliary benign diseases thus suggesting the role of this mediator in CCA pathogenesis [37]. The immunohistochemical analysis of 114 tissue specimens of CCA by the endothelial-specific antibody CD31 and the lymphoendothelial-specific antibody D2-40 demonstrated that an increased MVD is related to lower 5-year survival rates and to higher recurrence rates [19].

Platelet derived growth factor (PDGF), that plays a key role in blood vessel formation, resulted overexpressed in three CCA cell lines (OCA17, M156, and KKU100) and in human samples of CCA (84.6%). In the same study Authors observed that expression of PDGF positively correlates with stage, metastasis and short survival rate [38]. Another study, instead, demonstrated that the production of PDGF-D by CCA cells favors the recruitment of cancer-associated fibroblasts (CAFs) exerting a key role in the interplay between tumor and microenvironment. Particularly, PDGF-D promotes fibroblast migration through PDGFRβ and Rho GTPase and JNK activation [39].

CD146 is a cell adhesion molecule (CAM) that also exerts a role in cellular processes as signaling transduction, cell migration and angiogenesis [40]. In GBC patients, high CD146 expression correlated with high microvessel and lymphatic vessel counts, in particular in poorly differentiated adenocarcinoma, while peritumoral tissues, polyps, and chronic cholecystitis expressed lower levels of CD146 and had lower average microvessel and lymph vessel counts. Moreover, a high CD146 expression and a high vessel counts correlated with a lower OS of GBC patients suggesting their potential use as prognostic markers [41].

The last studies focus on the genomic landscape of BTC. Nakamura et al. characterized a large BTC cohort (260 cases, including iCCAs, eCCAs and GBC) of Japanese patients by a combination of whole-exome and transcriptome sequencing and uncovered molecular alterations. They identified 32 significantly altered genes, including potentially targetable genetic alterations in 40% of cases. FGFR2 fusion genes characterize iCCA cases while gene fusions involving the Protein Kinase CAMP-Activated Catalytic Subunit Alpha (PRKACA) or Protein Kinase CAMP-Activated Catalytic Subunit Beta (PRKACB) preferentially occurred in eCCA [42,43]. In GBC, inactivation of PTEN and TSC1 was frequently observed while EGFR family genes (EGFR, ERBB2 and ERBB3) were activated. Next generation sequencing led to the observation that KRAS mutations are characteristic of pCCA (22% to 53%), while isocitrate dehydrogenase (IDH)1/2 mutations are more common in iCCA [44,45]. Mutations of TP53 and SMAD4 have been observed in liver-fluke associated CCA while BAP1 and IDH1/2 were more often mutated in non-liver fluke associated CCA [46]. Furthermore, genomic studies confirmed that molecular pathways driving cell growth and angiogenesis, as EGF, RAS, AKT, and MET, are activated in BTCs [47] (Table 1). Lastly, the role of microRNAs (miR)s, small noncoding RNAs, has been suggested in the promotion of angiogenesis [48,49]. The only available data concern the miRNA101 that binds directly to the 3’-untranslated region (UTR) of both VEGF and COX-2 mRNAs as demonstrated by both computational analysis and experimental assays. miR-101 inhibits angiogenesis both in vitro and in vivo [50]*.* The incubation of human umbilical vein endothelial cells with the conditioned media collected from human CCA cells transduced with the miR-101 is associated to an anti-angiogenic effect when conditioned media from CCA cells overexpressing miR-10 were mixed with Matrigel and subcutaneously injected in C57BL/6 mice. 

**Figure 1:**
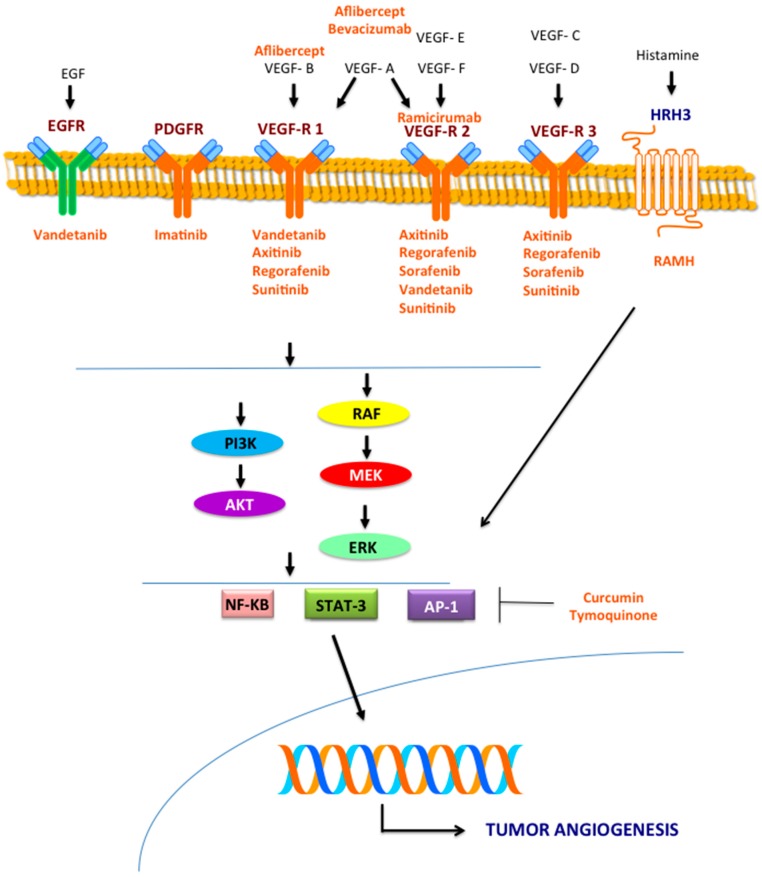
Mechanisms involved in the angiogenic process and the potential therapeutic role of drugs targeting the pro-angiogenic signaling pathways in tumors of the biliary tract.

Overall, these data suggest the existence of several molecules and mechanisms involved in the angiogenic process and the potential therapeutic role of drugs targeting the pro-angiogenic signaling pathways in tumors of the biliary tract (Figure 1).

## 3. Molecular Pathways Involved in Angiogenesis

### 3.1. VEGF Pathway

VEGF is the most relevant pro-angiogenic protein. Indeed, the signaling mediated from VEGF promote the proliferation, differentiation and migration of EC representing the pivotal mechanism both physiologically and in solid tumors [21]. The main stimulus for VEGF production is represented by hypoxia that leads to the expression of the transcription factor hypoxia inducible factor (HIF)1-α. Although tumor cells usually represent the major source of VEGF, tumor-associated stroma is also an important site of VEGF production [51]. The VEGF family is composed by six structurally related proteins that bind three different cell surface-expressed receptors (VEGFR-1/2/3). VEGFR-1 and -2 are mainly expressed on endothelial cells while VEGFR-3 in the lymphatic endothelium [52]. 

The major pro-angiogenic signal is generated from the ligand-activated VEGFR-2 that binds phospholipase (PL)-Cγ and activates protein-kinase C (PKC). This mechanism activates MAPK through a MEK-dependent mechanism and independently from RAS [53]. Other studies suggest that VEGF effect is also mediated by RAS activation that is essential for DNA synthesis both basally and after VEGF stimulation [54]. VEGFR-2 also activates phosphatidylinositol 3′-kinase (PI3K), which results in an increase of the lipid phosphatidylinositol (3,4,5)P_3_ and consequent activation of protein kinase B (Akt/PKB), endothelial nitric oxide synthase, and the small GTP-binding protein Rac [55].

### 3.2. MEK/ERK Pathway

The MEK/ERK pathway also exerts a pro-angiogenic effect in tumors and represents another potential target of anti-angiogenic drugs in BTCs. The role of this pathway in promoting angiogenesis is mainly related to the phosphorylation of eukaryotic translation initiation factor 4E-binding protein 1 (4E-BP1), S6-kinase (S6K), and MAP kinase interacting kinase (MNK) mediated by ERK. This process leads to an increased rate of mRNA translation into HIF-1α protein that is the main transcriptional inductor of several pro-angiogenic factors such as VEGF. ERK is also able to activate the transcription of HIF-1α by the co-activator CBP/p300 that increases HIF-1α/p300 complex formation [56].

### 3.3. Other Signaling Pathways

Another signaling pathway involved in the pathogenesis of CCA is the Yes-associated protein (YAP)/Hippo pathway. YAP regulates the expression of genes regulating proliferation, apoptosis and angiogenesis and is overexpressed in CCA cells. Particularly, YAP promotes the angiogenesis process interacting with TEAD transcription factors that have as transcriptional target the pro-angiogenic microfibrillar-associated protein 5 (MFAP5) [57]. CD31+ and MFAP5 expression was studied in xenograft models and was correlated to YAP activity [57]. 

NF-kB, signal transducer and activator of transcription (STAT)-3 and activator protein (AP)-1 also regulate the expression of various genes involved in proliferation, apoptosis, inflammation and angiogenesis [58,59] thus representing potential therapeutic targets of anti-angiogenic drugs. Particularly, STAT3, when constitutively activated, as in experimental models, causes an enhanced expression of VEGF [58].

Histamine also plays a role in regulating the cell growth in CCA. It acts via four receptors (HRH1, HRH2, HRH3, and HRH4) and, particularly, its binding to HRH3 is involved in CCA cancerogenesis interfering with VEGF signaling [60].

## 4. Preclinical Studies

### 4.1. Targeting the VEGF Pathway

*Bevacizumab*, a monoclonal antibody (mAb) neutralizing VEGF, was tested in a preclinical model of CCA, in which it exerted an anti-angiogenic effect through the inhibition of both the peri- and intra-tumoral vascularization resulting in the reduction of tumor growth. On the other side, bevacizumab led to an overexpression of HIF1α and HIF1α-responsive genes, involved in drug resistance mechanisms [61]. This is the only study exploring, in a preclinical setting, the anti-tumor effect of bevacizumab in BTC, although several clinical studies tested this drug in BTC, as reported below.

The multikinase inhibitor *sorafenib*, targeting VEGFR, platelet growth factor receptor (PDGFR) and RAF kinase, has been evaluated in a preclinical model of iCCA showing a relevant antitumor effect. Sorafenib inhibits the phosphorylation of MEK, MAPK and STAT3 while, in vivo, the oral administration of sorafenib inhibits the growth of subcutaneous tumors and prolongs the survival of mouse models with peritoneally disseminated disease [62]. Sorafenib-coated metal stents have been evaluated in vitro on HuCC-T1 cells and in vivo in a mouse xenograft model [63]. Cancer cells were seeded onto the polymer film and then allowed to proliferate. At a concentration higher than 10 mM the drug inhibited the tube formation of endothelial cells in vitro. Cell invasion also was completely inhibited at a dose of sorafenib higher than 25 mg/mL and migration was inhibited in a dose-dependent manner. The effects on both invasion and migration were maintained during the 30-day drug release experiment. The antitumor effect of sorafenib-coated stents was evaluated in vivo by using a mouse tumor xenograft model obtained by the subcutaneous injection of HuCC-T1 cells. When sorafenib-coated stents were placed under the tumor mass the growth of the tumor was reduced and the expression of molecules involved in apoptosis and mitosis signaling, such as B-cell lymphoma (Bcl)-2, Bcl-x, caspase-3 was significantly influenced by the sorafenib-coated stents [63].

*Vandetanib*, a VEGFR-2 and EGFR tyrosine kinase inhibitor, was tested in bioluminescent CCA cells and mouse xenograft models of CCA [64]. TKKK cell line, one of the four CCA cell lines investigated in this study, characterized by the highest expression of both EGFR and VEGF, was more sensitive to vandetanib [64]. In a subcutaneous xenograft model injected with TKKK cells, both the anti-tumor and anti-metastatic effect of vandetanib was demonstrated confirming the in vitro data [64] and suggesting that a particular setting of CCA, characterized by EGFR amplification and lack of K-RAS mutations, could benefit by the clinical employ of vandetanib. The immunohistochemical study of tumors from the TKKK xenografts showed a significant reduction of both MVD and Ki67 proliferation index. 

Another preclinical study evaluated the anti-tumor properties of *axitinib*, an oral specific VEGFR-1/2/3 tyrosine kinase inhibitor, in CCA. Authors screened eight CCA cell lines for expression of angiogenesis-related molecules by gene expression analysis and found that VEGF-A, VEGF-B, VEGF-C and VEGF-D were highly expressed in three cell lines [65]. Two of these cell lines were then used to obtain subcutaneous xenograft models and the MVD was studied to explore the anti-angiogenic effect of axitinib. This drug significantly reduced both the tumor growth and the neo-angiogenesis in xenograft models [65].

### 4.2. Targeting the MEK/ERK Pathway

An orthotopic model of GBC, obtained by inoculation of K-RAS mutated NOZ cells, was used to test the efficacy of the MEK inhibitor *U0126* [66]. The intraperitoneal injection of this drug significantly increased the survival of the mice orthotopically injected with NOZ cells, thus suggesting a new therapeutic strategy for advanced GBC. 

A combination therapy targeting both EGFR and MEK1/2 was tested in vitro and in vivo revealing a promising therapeutic strategy for biliary tract carcinoma. In vitro, the eCCA EGI-1, WITT cell lines and the iCCA MTCHC01 cell line were treated with *panitumumab*, *trametinib* or their combination. Trametinib was effective to inhibit MAPK-1 and -2 activation in all cell lines, characterized by different mutational status of K-RAS, while panitumumab was particularly able to reduce phospho-EGFR expression in K-RAS mutated EGI-1 cells and the MAPK signaling in K-RAS wild type WITT cells [67]. In vivo, the monotherapy with trametinib showed antitumor properties only in xenograft models subcutaneously injected with K-RAS mutated cells. The combination of trametinib with panitumumab did not potentiate the activity of trametinib in EGI-1 xenografts but the combination treatment was essential to slow the tumor growth in WITT xenografts, probably because this TKi is essential to overcome the resistance to panitumumab in the K-RAS wild type xenografts. The ant-iangiogenic effect was studied in vivo by the evaluation of CD31 expression and it was observed that trametinib and the combination therapy significantly reduced the CD31 expression in K-RAS mutated xenografts while in K-RAS wild type xenografts none of the treatments influenced the expression of this angiogenesis marker. However, the significant reduction of CD31 expression obtained by panitumumab alone in MT-CHC01 xenografts was associated to a paradoxical increased tumor growth. Consequently, the authors suggested that the anti-tumor and anti-angiogenic effect were independent, and that angiogenesis reduction was not enough to inhibit tumor growth [67].

### 4.3. Other Drugs

*Curcumin*, a natural phenol with anti-inflammatory properties, derived from turmeric (Curcuma longa), inhibits several transcription factors involved in CCA pathogenesis, such as NF-κB, signal transducer and activator of transcription (STAT)-3 and activator protein (AP)-1. Curcumin showed anti-proliferative and pro-apoptotic effects in CCA cell lines and decreased tumor development in a hamster model of CCA with higher survival rates. In this model the authors observed the anti-angiogenic properties of curcumin represented by the reduction of MVD and the suppression of VEGF expression, beyond the effects on cell proliferation, tumor invasion and apoptosis [68,69]. 

In a CCA xenograft mouse model, obtained by the subcutaneous injection of Mz-ChA-1 cells, the inhibition of histamine decarboxylase, that is responsible of histamine formation from histidine, downregulated the autocrine stimulation of histamine on tumor growth (~80%) and VEGF expression [70]. RAMH ((R)-(α)-(−)-methylhistaminedihydrobromide) is a HRH3 agonist and its antitumor effect was studied in vitro and in vivo. In vivo, RAMH inhibited tumor growth in Mz-ChA-1 xenografts and reduced the expression of VEGF-A, VEGF-C, VEGFR-2, and VEGFR-3 in tumor samples [71]. 

Lastly, *tymoquinone*, a derived from black cumin (*Nigella stativa*), exerts antitumor properties in cholangiocarcinoma models. In vitro and in vivo studies demonstrated that tymoquinone reduces the expression of COX-2, VEGF and cyclin B1, inhibiting the DNA-binding activity of NF-κB, thus reducing cell survival and proliferation [72].

These data suggest that the inhibition of angiogenesis has a good rationale in the therapeutic management of BTC. Indeed, the data are still lacking and several aspects, like those related to the drug resistance mechanisms, need to be clarified. Mechanisms of resistance are related both to cells of the microenvironment, as the myeloid cells that promote a VEGF-independent tumor angiogenesis [73], and to intrinsic tumor mechanisms, as the angiogenic signaling redundancy [74]. Moreover, although the anti-angiogenic therapy is promising, the recognition of markers of responsiveness or development of evasion to anti-angiogenic therapy is crucial for a personalized treatment of BTCs.

## 5. Clinical Studies in BTC Patients: Is There a Chance for a Phase III Trial?

Because of the relevance of angiogenesis in the development of BTCs and the data from preclinical studies, the activity of many anti-angiogenetic treatments with both antibodies (bevacizumab and ramucirumab) or trap (aflibercept) and TKIs (sorafenib, vandetanib, sunitinib, and regorafenib) were explored alone or in association to chemotherapy and/or anti-EGFR drugs in several phase I and II trials (Table 2).

### 5.1. Bevacizumab

In a phase II study, it was administrated with gemcitabine and oxaliplatin in 35 patients with untreated metastatic BTC with a favorable toxicity profile. Median progression free survival (PFS) was 7.0 months (95% CI 5.3–10.3), even if six-month PFS rate was 63% (47/79 patients), below the targeted rate of 70% [75]. Combination of bevacizumab with different chemotherapy and erlotinib (a TKI that inhibits EGFR) was evaluated in a multicenter phase II trial enrolling 49 patients. In this study, 12% and 51% of patients achieved a partial response and a stable disease, respectively. Median OS and TTP were 9.9 and 4.4 months, respectively, in the absence of severe adverse events. The Authors demonstrated that this combination might represent a therapeutic option in patients with metastatic BTC. However, the stratification performed for the molecular analysis of EGFR pathway showed that patients whose tumors had mutations in EGFR vIII, or had non-wild-type K-RAS may be less likely to respond to erlotinib treatment [76]. Bevacizumab was evaluated as second line therapy in association with FOLFIRI in 13 GEMOX pretreated metastatic (m)BTC patients. Five and six patients achieved partial response (PR) and stable disease (SD), respectively, with acceptable toxicities. Median PFS and mOS were 8 (95% CI: 7–16) and 20 (95% CI: 8–48) months, respectively. Response and disease control rates were 38.4% (95% CI: 12.5–89) and 84.5% (95% CI: 42–100), respectively [77]. An ongoing phase II randomized study will evaluate as primary end-point the survival and progression rates at six months in untreated advanced or metastatic K-RAS wild-type (WT) BTC patients treated with gemcitabine, oxaliplatin and capecitabine combination plus panitumumab or bevacizumab (GOC-B-P). Secondary end-point will be OS and response rate before cross-over; moreover, PFS and response rate will be evaluate after cross-over [78]. A phase II trial examined the activity of bevacizumab with FOLFOX-6 but the patients enrolled were too few to draw conclusions [79]. Another study evaluated the effect of bevacizumab with gemcitabine plus capecitabine combination [80] but results are not still available.

### 5.2. Ramucirumab

Activity of ramucirumab, a monoclonal anti-body direct against VEGFR 2, is actually being evaluated in a phase II study enrolling patients with pre-treated BTC. Primary outcome is PFS calculated as the time period from treatment start to disease progression or death, whichever occurs first, or to the last follow-up if no progression has being achieved [81]. A phase I trial is evaluating the safety and dose-limiting toxicities (DLT) for association of ramucirumab with pembrolizumab (an immunochekpoint inhibitor) in many cancer including BTC [82]. A phase II study is randomizing untreated advanced or metastatic BTC to receive ramucirumab or merestinib (a MET inhibitor) or placebo plus cisplatin/gemcitabine, with PFS as primary outcome measure, and with overall survival (OS), objective response rate (ORR), disease control rate (DCR), as main secondary endpoints [83].

### 5.3. Aflibercept

Aflibercept, a VEGF trap, is being tested in a phase I trial, associated with capecitabine in chemorefractory metastatic breast and digestive cancers, including BTC (MOMENTUM1). This study aims to assess the DLT and the maximum tolerated dose (MTD) in both metronomic or intermittent arms to define the recommended dose for the phase II trial [84].

### 5.4. Sorafenib

Sorafenib has been examined in several phase II trials in untreated patients with mBTC. Sorafenib monotherapy achieved disappointing results in two phase II trials. In the first, this TKI administered alone in 46 patients showed a disease control rate at 12 weeks of 32.6% with a mPFS and a mOS of 2.3 and 4.4 months, respectively, with an acceptable toxicity profile [85]. The second study, a phase II trial with sorafenib alone, was closed because the primary objective was not reached. The response rate was 0% (0%–11%), with 39% of patients with a SD. PFS and OS were three and nine months, respectively [86].

Combination therapy with chemotherapy and sorafenib or other TKIs was a failure. A double-blind phase II Working Group of Internal Oncology (AIO) study randomizing 102 patients with uresectable or metastatic BTC to gemcitabine with sorafenib or placebo did not showed an improved efficacy from the addition of the TKI. PFS and OS did not differ in two arms. A multivariate analysis showed that patients with liver metastasis and undergone resection of primary site survived longer if treated with sorafenib than placebo (*p =* 0.019). Moreover, in sorafenib arm, patients with hand-foot syndrome (HFS) showed longer PFS and OS than patients without HFS [87]. A phase II study evaluated the addition of sorafenib to gemcitabine and cisplatin in metastatic (m)BTC first-line therapy. Six-month PFS was 51%, with amPFS and mOS of 6.5 (95% CI: 3.5–8.3) and 14.4 months (95% CI: 11.6–19.2 months), respectively. All these data are worse compared to the historical achievement of platinum-based therapy, with an increased toxicity [88]. The phase II trial of Southwest Oncology Group (SWOG), which enrolled 34 patients with mBTC receiving sorafenib and erlotinib, was stopped because it failed to meet the requirement of at least 13 patients alive and without progression after four months from recruitment [91]. Actually, a phase I/II study of combination of gemcitabine and oxaliplatin (GEMOX) with sorafenib was concluded but the number of participants is too small to draw significant conclusions [90]. The combination of capecitabine and oxaliplatin with sorafenib was also studied in a phase I/II study but results are not still available [91]. An umbrella phase II trial evaluating several small molecules including sorafenib in association with GEMOX after the result of genomic and proteomic profiling of tumor tissue is actually ongoing for advanced or recurrent BTC [92].

### 5.5. Vandetanib

Vandetanib is a strong inhibitor of VEGFR-2 evaluated in one phase I trial and two large phase II trials. The maximum tolerate dose (MTD) and safety of vandetanib in association with gemcitabine and capecitabine have been evaluated in 23 mBTC patients. This combination was well tolerated with a recommended phase II dose of gemcitabine 1000 mg/m^2^ weekly for three consecutive weeks, capecitabine 850 mg/m^2^ BID dys 1–21, and vandetanib 300 mg daily, every 28 days [93]. In an Italian phase II trial, 173 patients with advanced BTC were randomized into three arms: vandetanib alone, vandetanib plus gemcitabine, and gemcitabine plus placebo. A mPFS of 105 (72–155), 114 (91–193) and 148 (71–225) days, respectively, was achieved without a statistical significant differences (*p* = 0.18), and with similar adverse events for the three groups [94]. 

### 5.6. Cediranib

In a multicenter phase II study, 124 patients were randomized to receive first-line cisplatin and gemcitabine chemotherapy with either 20 mg oral cediranib or placebo. Median PFS was 8.0 and 7.4 months, in cediranib and placebo group, respectively, without a statistical significance (HR 0.93; *p =* 0.72). Patients who received cediranib had more grade 3–4 toxic effects [95].

### 5.7. Sunitinib

An Asiatic phase II multicenter trial evaluated the activity of sunitinib, a multitarget TKI acting on angiogenesis, in 56 patients with pretreated mBTC. A marginal efficacy was observed since a median time to progression (TTP) of 1.7 months (95% CI 1.0–2.4) and a disease control rate of 50% was achieved [96]. In a report of three cases of patients with advanced ICC pretreated with standard chemotherapy, treatment with sunitinib achieved a sustained disease control superior to four months with either a PR or SD with a manageable side effect [102]. An ongoing single arm, phase II trial is evaluating mOS as primary end-point and PFS and ORR as secondary end-point in patients with advanced CCA treated with sunitib after a first line with chemotherapy [97].

### 5.8. Regorafenib

Regorafenib is another TKI that inhibits VEGFRs and TIE2. In a phase II trial, patients with mBTC that underwent no more than two prior lines of systemic chemotherapy are being enrolled to receive this drug. Primary end-point will be six-month OS rate with disease control rate and six-month PFS rate as secondary end-points [98]. In a similar phase II study, regorafenib is evaluated in advanced and metastatic BTC patients after the failure of a first-line chemotherapy. Primary and secondary outcomes were PFS, ORR, OS, and biomarkers changes, respectively [99]. Another study will analyze the combination of this TKI with GEMOX. Another ongoing phase Ib aims to identify the regorafenib dose associated to limiting toxicities during and within two cycles of treatment and to assess the right dose for the phase II study [100].

### 5.9. Selumetinib

The MEK1/2 inhibitor Selumetinib was evaluated in 28 patients with the response rate as primary endpoint. Sixty-eight percent of patients experienced a stable disease. Median PFS was 3.7 months while the median OS was 9.8 months. Rash (90%) and xerostomia (54%) were the most relevant side effects [101]. Another phase II study testing selumetinib with Cisplatin/Gemcitabine is recruiting patients and has as primary endpoint the change in tumor size [103].

### 5.10. Phase III Trials

Currently, there are neither closed nor ongoing posted phase III clinical trials testing anti-angiogenic drugs in mBTC. Considering that there are more negative than positive results from phase II non-randomized and randomized trials, we would consider far away a large-sample study. The failure to achieve the primary and secondary end-point may be due to the need to select population on the basis of cancer expression of the targets of these biological agents. In the future, the use of umbrella trials could be a turning point for the use of anti-angiogenic drugs in this malignancy.

## 6. Conclusions

Angiogenesis and lymphangiogenesis are crucial in the carcinogenesis of BTCs. Several studies confirmed an overexpression of molecules involved in the formation of new vessels on tumor samples and a correlation with a worse prognosis both in CCA and GBC. Alterations of genes involved in the angiogenic process, such as FGFR2, characterize these tumors and could interfere with the interplay between VEGF, TSP-1 and Ang-1/2. Different preclinical studies tested the effect of new anti-angiogenic drugs in BTCs with contrasting results, partially due to unknown drug resistance mechanisms. Furthermore, the role of new drugs in inhibiting the lymphangiogenesis has not been well elucidated and these aspects remain obscure. Moreover, phase I and II trials did not lead to encouraging results. Since BTCs include a large spectrum of different diseases, as confirmed from the recent genomic studies, these data suggest the need of trials enrolling selected patients. Indeed, the identification of patients with a hyperactivation of the pro-angiogenic pathways or with specific genic aberrations could identify a defined setting of patients that could benefit of the treatment with targeted therapies.

## Figures and Tables

**Table 1:** Genomic aberrations of biliary tract cancers (BTCs) linked to the angiogenesis and correlated to the location of tumors.

Subtype of BTC	Genomic Aberrations
iCCA	FGFR2 fusion genes
	IDH1/2 mutations
pCCA	KRAS mutations
eCCA	PRKACA and PRKACB fusion genes
GBC	PTEN (inactivated)
	TSC1 (inactivated)
	EGFR, ERBB2 and ERBB3 (activated)

**Table 2:** Clinical trials of anti-angionenic drugs in BTC.

Drug	Design	Regimen	Main Inclusion Criteria	Primary Outcomes	Status	Results	Reference
mAb	Phase II	Bevacizumab + GEMOX	Metastatic BTC	PFS	Terminated	mPFS: 7.0 months; PFS rate: 63%	[75]
mAb	Phase II	Bevacizumab + CT + erlotinib	Metastatic BTC	Response rate	Terminated	PR:12%; SD: 51%; mOS: 9.9 months; TTP: 4.4 months	[76]
mAb	Phase II	Bevacizumab + FOLFIRI	Second line therapy in GEMOX pretreated m-iCCA	Tolerance and efficacy	Terminated	PR: 5/13 pts; SD 6/13 pts; mPFS: 8 months; mOS: 20 months	[77]
mAb	Randomized phase II	Bevacizumab vs. panitumum + oxaliplatin/gemcitabine/capecitabine	Untreated advanced or metastatic K-RAS wild-type (WT) BTC	6-month survival rate; 6-month progression rates	Ongoing	Not achieved	[78]
mAb	Phase II	Bevacizumab + FOLFOX	Advanced BTC	Safely	Closed for slow accrual	Not drawn	[79]
mAb	Phase II	Bevacizumab + gemcitabine + capecitabine	Advanced or metastatic adenoca. of gallbladder or biliary ducts	Safety/efficacy	Ongoing	Not achieved	[80]
mAb	Phase II	Ramucirumab	Advanced, pre-treated BTCs	Safety/efficacy	Ongoing	Not achieved	[81]
mAb	Phase I	Ramucirumab + pembrolizumab	Metastatic BTC	Safety/DLT	Ongoing	Not achieved	[82]
mAb	Phase II	Ramucirumab vs. merestinib + cisplatin/Gemcitabine	Advanced or metastatic BTC	PFS	Ongoing	Not achieved	[83]
VEGF trap	Phase I	Aflibercept + capecitabine	Chemorefractory metastatic BTC	DLT/MTD	On going	Not achieved	[84]
TKI	Phase II	Sorafenib	Advanced BTC	Disease control rate at 12 weeks	Terminated	Disease control rate at 12 weeks: 32.6%; mPFS: 2.3 months; mOS: 4.4 months	[85]
TKI	Phase II	Sorafenib	Advanced BTC	Objective response rate	Closed for not achieved primary objective	Response rate: 0%; SD: 39%; PFS: 3 months; OS: 9 months	[86]
TKI	Double-blind randomized phase II	Sorafenib + gemcitabine vs. placebo + gemcitabine	Unresectable or metastatic mBTC	PFS	Terminated	PFS: 4.9 vs. 3.0 months (*p* = 0.859); mOS: 11.2 vs. 8.4 months (*p* = 0.775)	[87]
TKI	Phase II	Sorafenib + gemcitabine/cisplatin	mBTC first-line therapy	6-month PFS	Terminated	6-month PFS: 51%, mPFS: 6.5 months; mOS: 14.4 months	[88]
TKI	Phase II	Sorafenib + erlotinib	mBTC first-line therapy	PFS	Stopped for failure to meet the main requirement	Unconfirmed PR: 2/13; mPFS: 2 months; mOS: 6 months	[89]
TKI	Phase I/II	Sorafenib + GEMOX	Advanced BTC	Safely	Closed for slow accrual	Not drawn	[90]
TKI	Phase I/II	Sorafenib + capecitabine/Oxaliplatine	Advanced BTC	Safety/efficacy	Ongoing	Not achieved	[91]
TKI	Umbrella phase I/II trial	Sorafenib vs. other TKI + GEMOX	Advanced or metastatic GBC or eCCA	Safety/efficacy	Ongoing	Not achieved	[92]
TKI	Phase I	Vandetanib + gemcitabine/capecitabine	mBTC	MTD/safety	Terminated	Vandetanib 300 mg daily; good safety profile	[93]
TKI	Randomized phase II	Vandetanib vs. vandetanib + gemcitabine vs. gemcitabine	Advanced BTC	mPFS	Terminated	mPFS: 105 vs. 114 vs. 148 days (*p =* 0.18)	[94]
TKI	Randomized phase II	Cediranib vs. placebo + cisplatin/gemcitabine	Advanced BTC	mPFS	Terminated	mPFS: 8 vs. 7.4 months (*p =* 0.72)	[95]
TKI	Phase II	Sunitinib	Second line unresectable, metastatic BTC	Safety/efficacy	Terminated	mTTP: 1.7 months; Objective RR: 8.9%; Grade 3–4 toxicities in 46.4% of patients	[96]
TKI	Phase II	Sunitinib	Advanced CCA	PFS/ORR	Ongoing	Not achieved	[97]
TKI	Phase II	Regorafenib	mBTC with no more than 2 prior lines CT	6-month OS rate	Ongoing	Not achieved	[98]
TKI	Phase II	Regorafenib	advanced, metastatic BTC, after first-line CT	PFS	Ongoing	Not achieved	[99]
TKI	Phase Ib/II	Regorafenib + GEMOX	advanced BTC	MTD/safety	Ongoing	Not achieved	[100]
TKI	Phase II	Selumetinib	metastatic BTC	PFS	Terminated	mPFS: 3.7 months	[101]

GEMOX: gemcitabine/oxaliplatin; FOLFOX: folinic acid/fluorouracil/oxaliplatin; CT: chemotherapy; TKI: tyrosine kinase inhibitor; mPFS: median progression free survival; PFS: progression free survival; DLT: dose limiting toxicity; OS: overall survival; PR: partial response; SD: stable disease; CCA: cholangiocarcinoma; MTD: maximum tolerated dose.
